# Data on the genome of *Bacillus subtilis* A1- Midalam from beach soil

**DOI:** 10.1016/j.dib.2021.107552

**Published:** 2021-11-07

**Authors:** Sneha Pramod, Rhea Thomas Thommana, Harini Kulanthaivelu Kanagam, Ashmita Suresh Kumar, Santha Kalaikumari S, Elavarashi Elangovan, Kumar Perumal

**Affiliations:** a Department of Biotechnology, Sri Ramachandra Institute of Higher Education and Research, Porur, Tamilnadu, India; b Department of Food and Bioproduct Sciences, College of Agriculture and Bioresources, University of Saskatchewan, Canada; c College of science, Northeastern University, 360 Huntington Ave, Boston, Massachusetts 02115, USA; d Department of Genetics, School of Biological Sciences, Madurai Kamaraj University, Madurai, India; e Biochemistry and Molecular Biology Department, Johns Hopkins University, Bloomberg School of Public Health, Baltimore, USA; f Department of Cancer Biology, Wake Forest School of Medicine, North Carolina, USA

**Keywords:** Genome sequencing, Biofilm, Antimicrobial peptide, Phylogenetic analysis

## Abstract

The draft genome sequence of *Bacillus subtilis* A1, isolated from beach soil, has been shown to produce biofilm. The genome size is 4,215,114 bp with an average G+C content of 43.5%. The genome of Bacillus subtilis A1 has 4413 total genes which include 4166 protein-coding sequences, 126 pseudo genes, 10 rRNA genes with 3 operons (5S, 16S and 23S), 86 tRNA genes and 5 noncoding RNA (ncRNA) genes. The genome contains genes coding for surfactin, fengycin, bacillaene, sublancin 168, bacillibactin, subtilosin A, bacilysin. The whole genome project has been deposited in GenBank under the accession number CP075344.1. The raw data is available at https://www.ncbi.nlm.nih.gov/nuccore/CP075344.1.

## Specifications Table

**Table:** 

Subject	Microbiology
Specific subject area	Genomics
Type of data	Figures, Table, genome sequencing data is in FASTA format
How data were acquired	Genome was sequenced with Illumina Hiseq-X sequencing at AgriGenome (Kerala, India)
Data format	Raw and analysed data sequence
Parameters for data collection	The pure culture of Bacillus subtilis A1 was used to isolate genomic DNA. Further genomic DNA was sequenced and analysed.
Description of data collection	Genomic DNA extraction was carried out using SDS and ultrasonic lysis and sequenced by Illumina Hiseq X. The adaptor sequences and low quality bases are trimmed using AdapterRemoval-v2 (version 2.3.1). The pre-processed reads are aligned to the reference genome NC_000964.3. The alignment is performed using the BWA MEM Program. Genome annotation was carried out using NCBI Genome Automatic Annotation Pipeline (PGAP)
Data source location	*Bacillus subtilis* A1 was isolated from Beach soil (Midalam, Tamil Nadu)
Data accessibility	The sequence data has been deposited at GenBank with the BioProject number: PRJNA729632, BioSample number: SAMN19136531 under the accession number CP075344.1 (https://www.ncbi.nlm.nih.gov/nuccore/CP075344.1). The SRA records could be accessed (https://www.ncbi.nlm.nih.gov/sra/PRJNA729632).

## Value of the Data

• The genome data of strain *Bacillus subtilis* A-1 helps to identify genes related to biofilm and used for discovering mechanism involved in quorum sensing.
• Comparison of the whole genome sequencing data of the strain *Bacillus subtilis* A-1 with that of other *Bacillus subtilis* strains might provide wide knowledge on the enzymes and its production mechanism.
• It also helps to understand the genetic structure and production of secondary metabolites especially non-ribosomal peptides which might provide pharmaceutical implications.
• Comparative genomics might provide knowledge on identifying genes with respect to metal resistance since the bacterium is isolated from metal rich environment.

## 1 Data Description

**Fig. 1:**
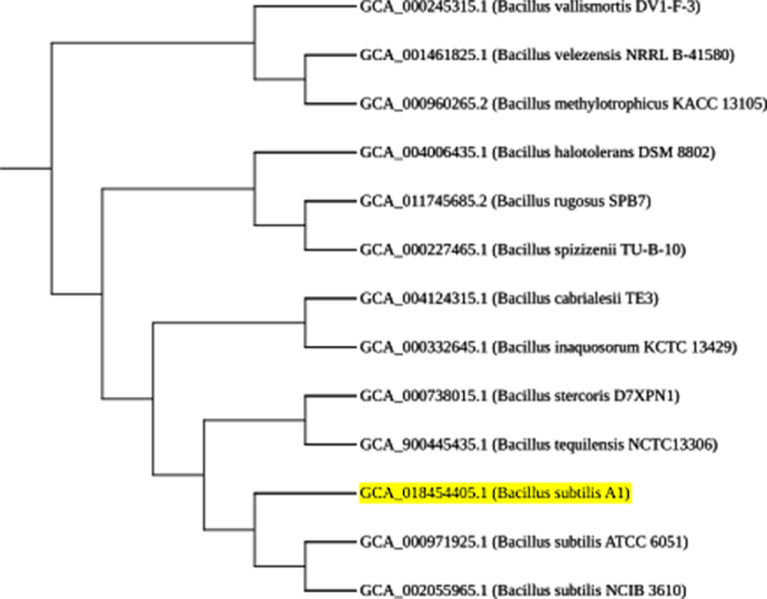
Phylogenetic relationship of 16s sequence of *Bacillus subtilis* A1.

**Fig. 2:**
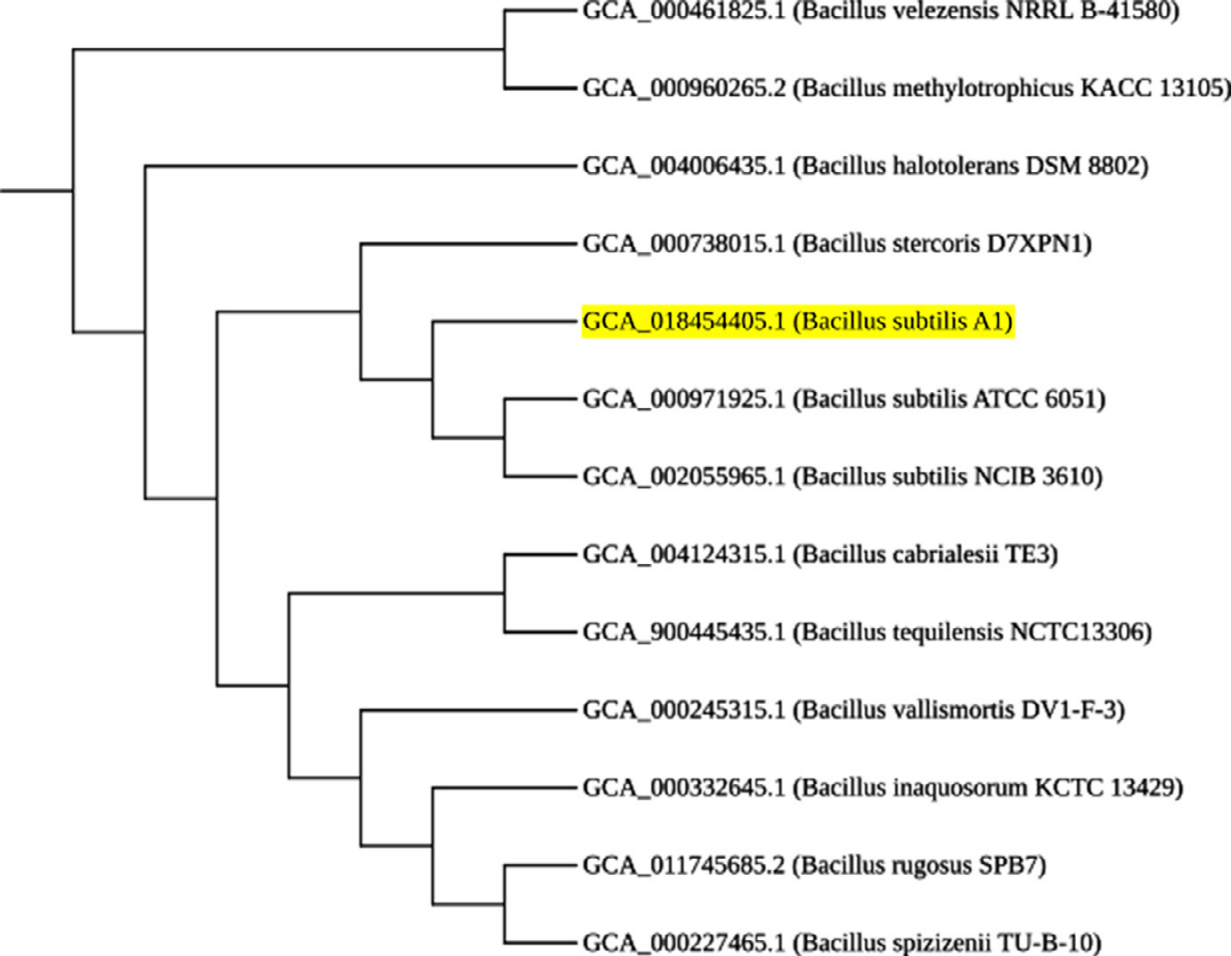
Phylogenetic relationship of whole genome sequencing from *Bacillus subtilis* A1.

**Fig. 3:**
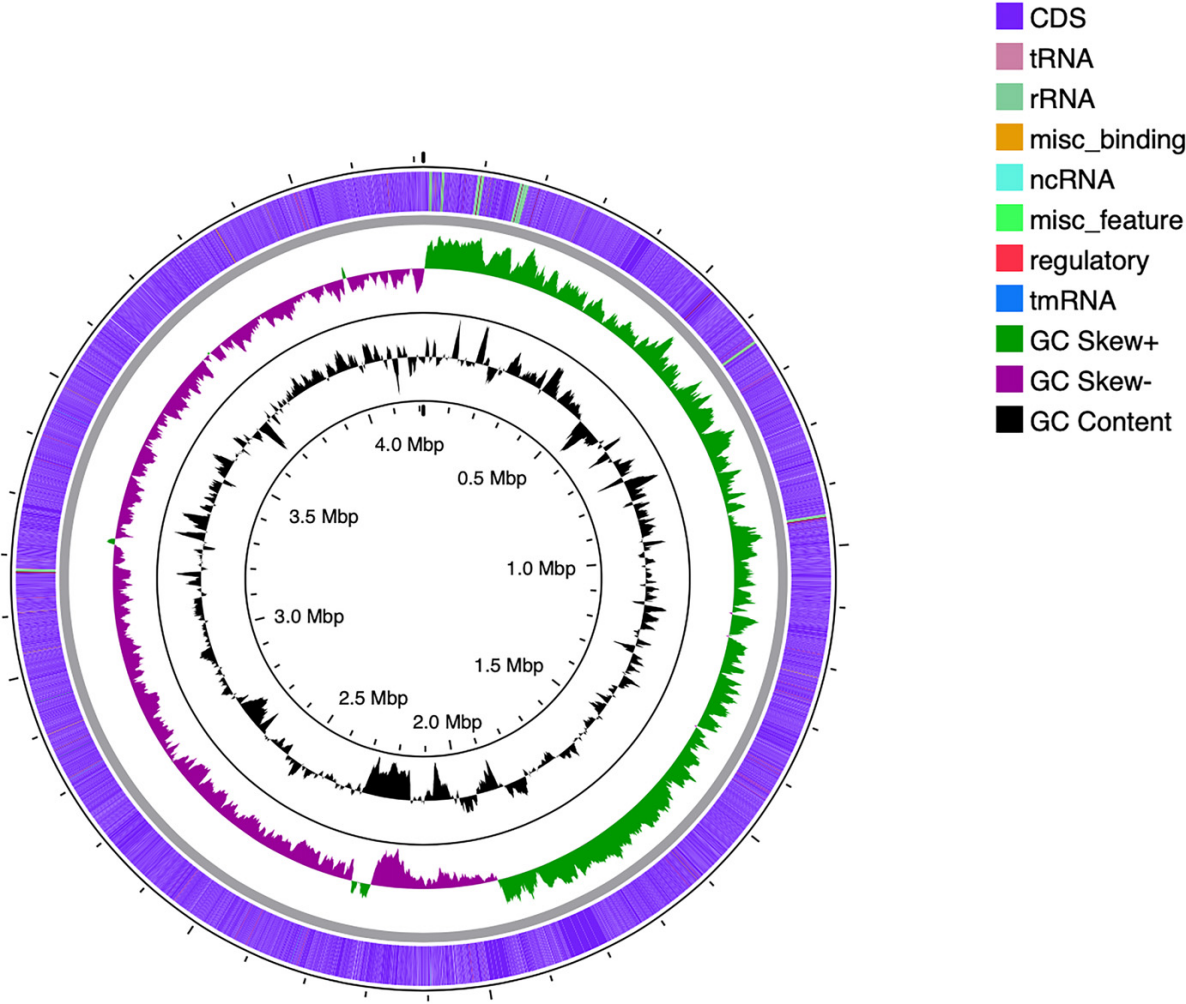
Genomic organisation of *Bacillus subtilis* A1- Midalam.

**Fig. 4:**
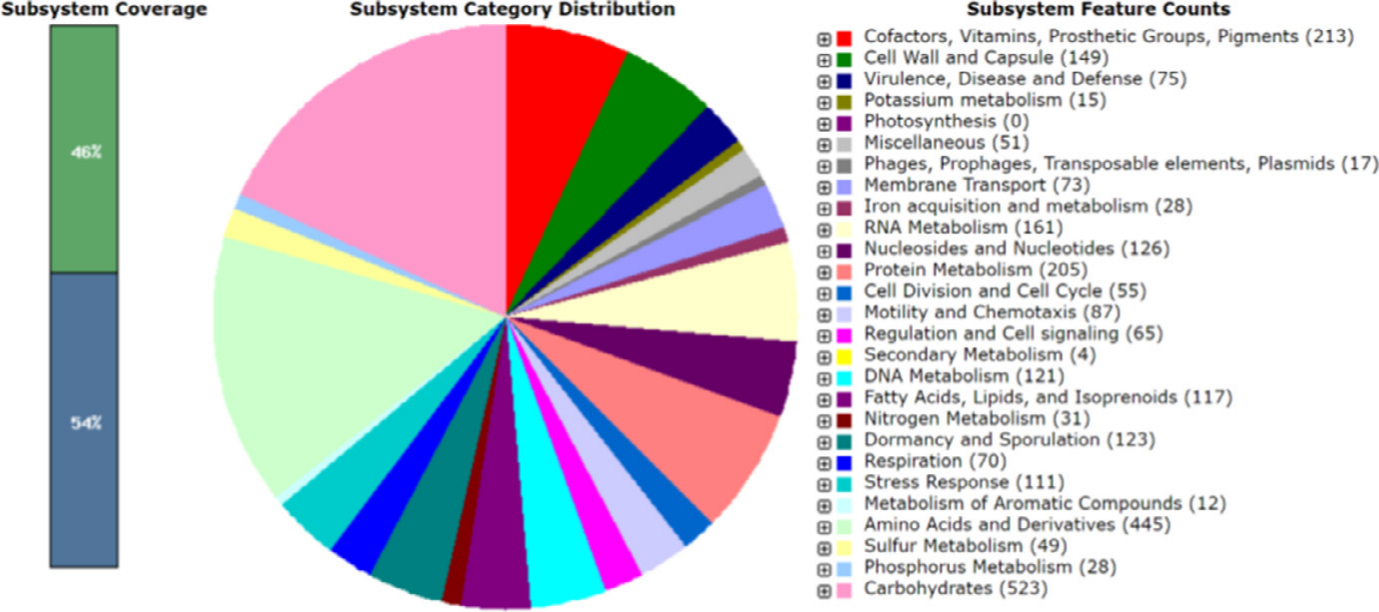
The whole genome sequence of the strain *Bacillus subtilis* A1 was annotated using the Rapid Annotation System Technology (RAST) server. The pie chart demonstrates the subsystem category distribution.

**Fig. 5:**
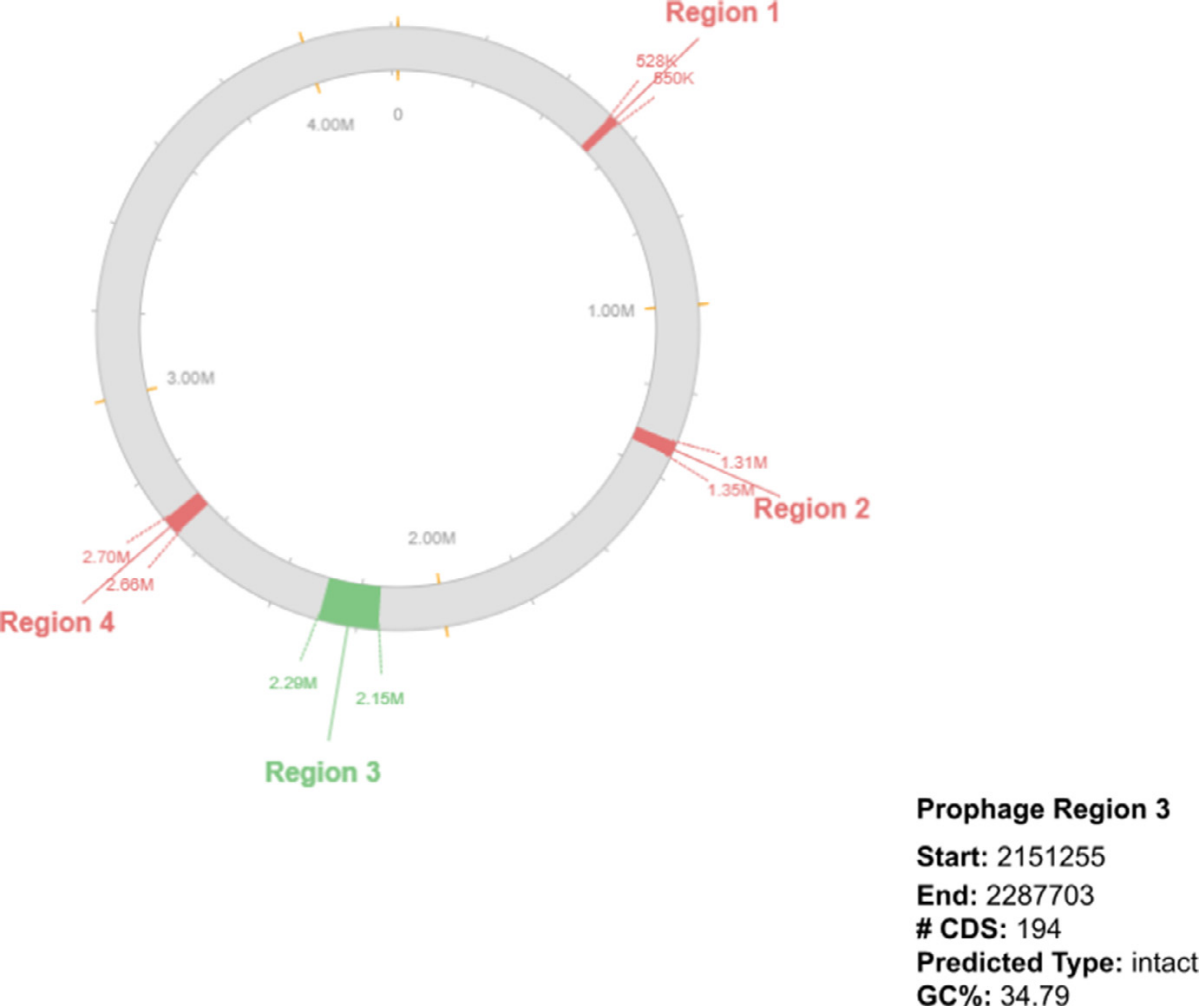
Prophage regions of *Bacillus subtilis* A1.

*Bacillus subtilis* is a Gram positive, catalase positive, non-pathogenic bacteria and is one of the most commonly studied bacterial species and used for producing several proteins and the organism is considered generally as safe (GRAS). It is a rod-shaped bacterium also called hay or grass bacillus, and capable of forming stress resistant endospores. They form biofilms and are sensitive to most antibiotics [1]. *Bacillus subtilis* also yields a variety of secondary metabolites that can be advantageous for commercial extraction and use. These include lipopeptides (surfactin, fengycin, iturin etc.), and RiPPs (lanthipeptides, thiopeptides etc.) among others. These metabolites have antimicrobial features that enhance its function as a biocontrol agent. Specifically, fengycin and surfactin are antibacterial and antifungal agents. Additionally, lanthipeptides and sactipeptides are commonly used as antibiotics [2]. In this study, the organism was isolated from beach soil and characterized using 16s rRNA sequencing and the sequence was submitted with MT361322.1. Fig. 1 represents the phylogenetic analysis of 16s sequences of isolates showing close relativity with the strain *Bacillus subtilis*. The whole genome sequencing of the isolate *Bacillus subtilis* A1 showing the evolutionary history was inferred and revealed close proximity to the strains of *Bacillus subtilis* (Fig. 2). Whole genome sequencing analysis along with genome annotation was performed and the assembled sequence was submitted in Gen bank with the accession id CP075344.1. The genome annotation features are provided in Table 1. The draft genome contains single contig with 4215114 bp. The genome of *Bacillus subtilis* A1 has 4413 total genes which include 4166 protein-coding sequences, 126 Pseudo genes, 10 rRNA genes with 3 operons (5S, 16S and 23S), 86 tRNA genes and 5 noncoding RNA (ncRNA) genes. A genomic circular map is provided in Fig. 3. The taxonomic position of the strain A1 was determined by multilocus sequence typing (MLST), using internal fragments of seven genes including purH, glpF, pycA, ilvD, rpoD, tpiA and pta. The RAST server identified the genome sequence of size 4,215,114 to have 4553 features, comprising 4437 coding sequences and 116 RNAs (5S RNA - 10, LSU rRNA - 10, SSU rRNA - 10, tRNA - 86). The sequence has GC content of 43.5% and 476 subsystems which is represented in Fig. 4. The presence of prophage sequences in the *Bacillus* genome A1 was analyzed and identified four prophage regions, of which 1 region was intact, 3 regions were incomplete (Fig. 5). Intact regions of prophages were located between positions 2151255 and 2287703 bp of length 136.4 Kb with total proteins of 194 is highlighted in Fig. 5. The strain A1 codes for major facilitator superfamily (MFS) antibiotic efflux pump and it codes for virulence factors mph (k) coding for spiramycin, telithromycin resistance, gene aadk coding for streptomycin and a gene tet (L) coding for doxycycline and tetracycline. No plasmids were found in the isolated genome. The genome contains genes responsible for the production of several bioactive secondary metabolites. The organism codes for surfactin, Bacillaen, fengycin, Sublancin 168, Bacillibactin, Subtilosin A and Bacilysin. The details of the secondary metabolites are provided in Table 2. The variant data was annotated using snpEff (supplementary file 1).

**Table 1:** General genomic feature of *Bacillus subtilis* A1.

Attributes	Value
Genome size (bp)	4,215,114
Genes (total)	4413
CDSs (total)	4292
Genes (coding)	4166
Genes (RNA)	121
rRNAs	10
tRNAs	86
ncRNAs	5
Pseudo Genes (total)	126

**Table 2:** List of Antimicrobial Peptides in *Bacillus subtilis* A1.

Type	From	To	Most similar known cluster	Similarity(%)
Ranthipeptide, sactipeptide	204175	226248	Sporulation killing factor	100
NRPS	358303	421744	Surfactin	82
TransAT-PKS, PKS-like, T3PKS, transAT-PKS-like, NRPS	1763763	1878521	Bacillaene	100
NRPS, betalactone	1935448	2017660	Fengycin	100
Glycocin	2259521	2279691	Sublancin 168	100
NRPS	3260519	3310260	Bacillibactin	100
Sactipeptide	3826058	3847669	Subtilosin A	100
Other	3850668	3892086	Bacilysin	100

## 2 Experimental Design, Materials and Methods

### 2.1 Phylogenetic analysis

The organism was isolated from beach soil and characterized by PCR using 16s rRNA sequencing and whole genome sequencing of the isolates. The evolutionary history was inferred using TYGS tool [3] to reveal a close proximity to other strains.

### 2.2 Extraction of DNA and whole genome sequencing

A single colony of *Bacillus spp* was cultivated in Luria- Bertani medium at 37 ° C overnight. The bacterial cells were lysed using ultrasonic [4] and pelleted using a centrifuge for the extraction of DNA and sequenced at AgriGenome Labs, Kochi, Kerala. Sequencing was performed using Illumina HiSeq X. The library preparation was carried out using NEBNext Ultra DNA Library Prep kit. The reads are generated R1=3,175,214 and R2= 3,175,214 with a mean length of 150 bp. The raw reads (.fastq) of the sequenced genome were subjected to pre-processing. Low quality reads were trimmed using Adapter Removal v-2. The sample genome was compared to the reference strain 168 (lab strain of *Bacillus subtilis*), using BWA MEM program. Once genome assembly was complete, the aligned reads were sorted and duplicates were removed using sambamba (v-0.8.0). Bcftools was utilized to assess Single Nucleotide Polymorphisms (SNPs) and Indels. The taxonomic position of strain A1 was determined by the MLST database (PubMLST; http://pubmlst.org/bsubtilis/) [5]. Genes were predicted by NCBI Prokaryotic Genome annotation pipeline (PGAP) [6]. Annotation was performed with RAST [7] using the RASTtk scheme [8]. Functional analysis was carried out using the tools available in SEED portal [9] with phaster [10]. Antibiotic resistance was determined using CARD [11]. The genome was screened for the presence of plasmids using PlasmidFinder 2.1 [12]. Several bioactive secondary metabolites were revealed by antiSMASH [13].

## Ethics Approval

CSP/20/FEB/84/97

## CRediT Author Statement

**Sneha Pramod:** Writing – original draft, Formal analysis; **Rhea Thomas Thommana:** Writing – original draft, Software, Formal analysis; **Harini Kulanthaivelu Kanagam:** Writing – original draft, Software, Formal analysis; **Ashmita Suresh Kumar:** Writing – original draft, Formal analysis; **Santha Kalaikumari S:** Visualization, Writing – review & editing; **Elavarashi Elangovan:** Writing – review & editing, Supervision; **Kumar Perumal:** Writing – review & editing, Supervision.

## Declaration of Competing Interest

The authors declare that they have no known competing financial interests or personal relationships, which have, or could be perceived to have, influenced the work reported in this article.

## Appendix Supplementary materials

**Supplementary file 1.** Variant data of *Bacillus subtilis* A1 Image, application 1
